# Chlorido(dimethyl sulfoxide)(pyridine-2-thiol­ato *N*-oxide-κ^2^ *S*,*O*)platinum(II)

**DOI:** 10.1107/S1600536808019041

**Published:** 2008-06-28

**Authors:** B. Ravindran Durai Nayagam, Samuel Robinson Jebas, P. Selvarathy Grace, Dieter Schollmeyer

**Affiliations:** a Department of Chemistry, Popes College, Sawyerpuram 628 251, Tamilnadu, India; b Department of Physics, Karunya University, Karunya Nagar, Coimbatore 641 114, India; c Institut für Organische Chemie, Universität Mainz, Duesbergweg 10-14, 55099 Mainz, Germany

## Abstract

The asymmetric unit of the title compound, [Pt(C_5_H_4_NOS)Cl(C_2_H_6_OS)], contains two independent complex mol­ecules having similar geometries. Each Pt^II^ atom is four-coordinated in a distorted square-planar geometry by S and O atoms of one pyridine *N*-oxide ligand, the S atom of one dimethyl sulfoxide mol­ecule and one terminal Cl^−^ ion. The mol­ecules are linked into a three-dimensional framework by C—H⋯O and C—H⋯Cl hydrogen bonds.

## Related literature

For biological activities of platinum, see: Weiss & Christian (1993 ▶); Loehrer *et al.* (1988 ▶); For biological activities of *N*-oxide derivatives, see: Bovin *et al.* (1992 ▶); Katsuyuki *et al.* (1991 ▶); Leonard *et al.* (1955 ▶); Lobana & Bhatia (1989 ▶); Symons & West (1985 ▶). For related literature, see: Jebas *et al.* (2005 ▶); Ravindran *et al.* (2008 ▶); Dyksterhouse *et al.* (2000 ▶); Ohms *et al. *(1982 ▶); Ravindran *et al. *(2008 ▶). For bond-length data, see: Allen *et al.* (1987 ▶).
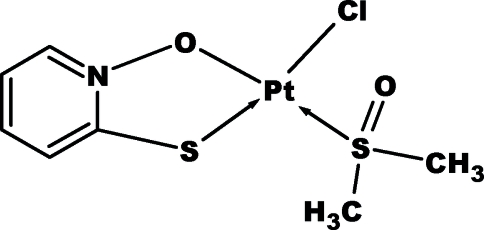


## Experimental

### 

#### Crystal data

[Pt(C_5_H_4_NOS)Cl(C_2_H_6_OS)]
*M* *_r_* = 434.82
Triclinic, 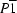

*a* = 10.2407 (5) Å
*b* = 10.9703 (5) Å
*c* = 10.9772 (6) Å
α = 82.950 (1)°
β = 76.720 (1)°
γ = 76.554 (1)°
*V* = 1164.21 (10) Å^3^
*Z* = 4
Mo *K*α radiation
μ = 12.61 mm^−1^
*T* = 173 (2) K
0.47 × 0.31 × 0.15 mm

#### Data collection

Bruker Kappa APEXII area-detector diffractometer
Absorption correction: Gaussian (Coppens, 1970 ▶) *T*_min_ = 0.5, *T*_max_ = 1.0
22889 measured reflections
5542 independent reflections
5032 reflections with *I* > 2σ(*I*)
*R*_int_ = 0.034

#### Refinement

*R*[*F* ^2^ > 2σ(*F* ^2^)] = 0.028
*wR*(*F* ^2^) = 0.070
*S* = 1.03
5542 reflections
257 parameters
H-atom parameters constrained
Δρ_max_ = 5.99 e Å^−3^
Δρ_min_ = −1.02 e Å^−3^

### 

Data collection: *APEX2* (Bruker, 2006 ▶); cell refinement: *APEX2*; data reduction: *SAINT* (Bruker, 2006 ▶); program(s) used to solve structure: *SHELXS97* (Sheldrick, 2008 ▶); program(s) used to refine structure: *SHELXL97* (Sheldrick, 2008 ▶); molecular graphics: *SHELXTL* (Sheldrick, 2008 ▶); software used to prepare material for publication: *SHELXTL* and *PLATON* (Spek, 2003 ▶).

## Supplementary Material

Crystal structure: contains datablocks global, I. DOI: 10.1107/S1600536808019041/ci2615sup1.cif

Structure factors: contains datablocks I. DOI: 10.1107/S1600536808019041/ci2615Isup2.hkl

Additional supplementary materials:  crystallographic information; 3D view; checkCIF report

## Figures and Tables

**Table:** 

Pt1—O7	2.020 (4)
Pt1—S2	2.1826 (13)
Pt1—S1	2.2495 (15)
Pt1—Cl1	2.3461 (13)
Pt2—O17	2.005 (4)
Pt2—S4	2.1850 (14)
Pt2—S3	2.2543 (14)
Pt2—Cl2	2.3402 (14)

**Table:** 

O7—Pt1—S2	179.05 (12)
O7—Pt1—S1	86.29 (11)
S2—Pt1—S1	93.15 (5)
O7—Pt1—Cl1	89.02 (11)
S2—Pt1—Cl1	91.57 (5)
S1—Pt1—Cl1	174.53 (5)
O17—Pt2—S4	179.35 (11)
O17—Pt2—S3	86.41 (11)
S4—Pt2—S3	94.02 (5)
O17—Pt2—Cl2	87.69 (11)
S4—Pt2—Cl2	91.89 (5)
S3—Pt2—Cl2	174.07 (5)

**Table 2:** Hydrogen-bond geometry (Å, °)

*D*—H⋯*A*	*D*—H	H⋯*A*	*D*⋯*A*	*D*—H⋯*A*
C9—H9*A*⋯O17^i^	0.98	2.43	3.351 (8)	157
C9—H9*B*⋯Cl1	0.98	2.73	3.350 (6)	121
C10—H10*A*⋯Cl1^ii^	0.98	2.73	3.601 (7)	149
C13—H13⋯O7^iii^	0.95	2.37	3.268 (8)	158
C15—H15⋯O8^iv^	0.95	2.39	3.271 (7)	155
C19—H19*A*⋯O8^v^	0.98	2.54	3.456 (7)	155
C20—H20*A*⋯O18^vi^	0.98	2.49	3.460 (8)	172
C20—H20*B*⋯Cl2	0.98	2.75	3.366 (7)	122

Symmetry codes: (i) 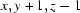; (ii) 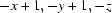; (iii) 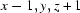; (iv) 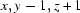; (v) 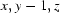; (vi) 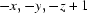.

**Fig. 1.**
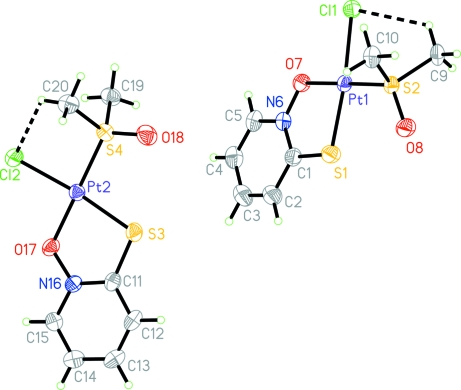
The asymmetric unit of the title compound, showing the atomic numbering scheme. Displacement ellipsoids are drawn at the 50% probability level. Hydrogen bonds are shown as dashed lines.

**Fig. 2.**
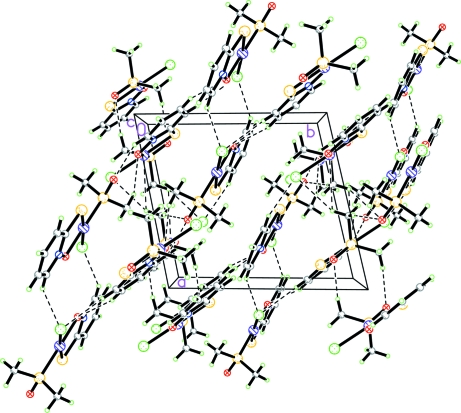
The crystal packing of the title compound, viewed along the c axis. Hydrogen bonds are shown as dashed lines.

## supplementary crystallographic information

### Comment

The platinum complex, *cis*-diamminedichloroplatinum(II) (cisplatin) is one of the most widely used antitumor drugs in the world (Weiss & Christian, 1993; Loehrer *et al.*, 1988). N-Oxides and their derivatives show a broad spectrum of biological activity, such as antifungal, antibacterial, antimicrobial and antibacterial activities (Lobana & Bhatia, 1989; Symons *et al.*, 1985). These compounds are also found to be involved in DNA strand scission under physiological conditions (Katsuyuki *et al.*, 1991; Bovin *et al.*, 1992). Pyridine N-oxides bearing a sulfur group in position 2 display significant antimicrobial activity (Leonard *et al.*, 1955). In view of the importance of the metal platinum and N-oxides, we have previously reported the crystal structures of N-oxide derivatives (Jebas *et al.*, 2005; Ravindran *et al.*, 2008). As an extension of our work on these derivatives, we report here the crystal structure of the title compound (Fig. 1).

The asymmetric unit of the title compound contains two independent complex molecules having similar geometries (Fig. 1 and Table 1). Each Pt^II^ atom is four-coordinated in a distorted square-planar geometry by S and O atoms of one pyridine N-oxide ligand, S atom of one dimethyl sulfoxide molecule and one terminal Cl^-^ ion. The average Pt—O [2.013 (4) Å] and Pt—S [2.218 (14) Å] distances are comparable with the values reported in the literature (Dyksterhouse *et al.*, 2000; Ravindran *et al.*, 2008). The mean C—S bond distance [1.755 (6) Å] is slightly longer than that reported for the uncoordinated thione molecule [1.692 (2)–1.698 (2) Å; Ohms *et al.*,1982]. The N—O bond length is in good agreement with the mean value of 1.304 (15) Å reported in the literature for N-oxides (Allen *et al.*, 1987). The dihedral angle between the two pyridine rings is 26.0 (3)°. The dihedral angle between the Pt1/S1/C1/N6/O7 and N6/C1-C5 planes is 0.6 (2)° and that between the Pt2/S3/C11/N16/O17 and N16/C11-C15 planes is 4.4 (2) Å, respectively.

The crystal packing is stabilized by intermolecular C—H··· O and C—H···Cl hydrogen bonding (Fig. 2).

### Experimental

A mixture of 2-benzylsulfanyl pyridine N-oxide, (0.219 g 1 mmol) and potassium tetrachloroplatinate(II) (0.415 g, 1 mmol) in water (20 ml) and methanol (20 ml) was heated at 333 K with stirring for 30 min. A yellow colour mass formed was dissolved in DMSO (10 ml) and kept at 278 K for a week. The compound formed was filtered off and dried. The compound was dissolved in chloroform and allowed to undergo slow evaporation. Fine crystals were obtained after a week.

### Refinement

H atoms were positioned geometrically [C-H = 0.95 (aromatic) or 0.98 Å (methyl)] and refined using a riding model, with *U*_iso_(H) = 1.2-1.5*U*_eq_(C). A rotating group model was used for the methyl groups. The highest residual density peak is located 1.15 Å from atom Pt2 and the deepest hole is located 0.92 Å from atom Pt1.

### Crystal data

**Table:**

[Pt(C_5_H_4_NOS)Cl(C_2_H_6_OS)]	*Z* = 4
*M**_r_* = 434.82	*F*_000_ = 808
Triclinic, *P*1	*D*_x_ = 2.481 Mg m^−^^3^
Hall symbol: -P 1	Mo *K*α radiation λ = 0.71073 Å
*a* = 10.2407 (5) Å	Cell parameters from 9909 reflections
*b* = 10.9703 (5) Å	θ = 2.5–27.8º
*c* = 10.9772 (6) Å	µ = 12.61 mm^−^^1^
α = 82.950 (1)º	*T* = 173 (2) K
β = 76.720 (1)º	Plate, colourless
γ = 76.554 (1)º	0.47 × 0.31 × 0.15 mm
*V* = 1164.21 (10) Å^3^	

### Data collection

**Table:**

Bruker Kappa APEXII area-detector diffractometer	5032 reflections with *I* > 2σ(*I*)
Monochromator: graphite	*R*_int_ = 0.034
*T* = 173(2) K	θ_max_ = 27.9º
ω and φ scans	θ_min_ = 1.9º
Absorption correction: Gaussian(Coppens, 1970)	*h* = −13→13
*T*_min_ = 0.5, *T*_max_ = 1.0	*k* = −14→14
22889 measured reflections	*l* = −14→14
5542 independent reflections	

### Refinement

**Table:**

Refinement on *F*^2^	Secondary atom site location: difference Fourier map
Least-squares matrix: full	Hydrogen site location: inferred from neighbouring sites
*R*[*F*^2^ > 2σ(*F*^2^)] = 0.028	H-atom parameters constrained
*wR*(*F*^2^) = 0.070	*w* = 1/[σ^2^(*F*_o_^2^) + (0.0265*P*)^2^ + 7.1392*P*] where *P* = (*F*_o_^2^ + 2*F*_c_^2^)/3
*S* = 1.03	(Δ/σ)_max_ = 0.001
5542 reflections	Δρ_max_ = 5.99 e Å^−^^3^
257 parameters	Δρ_min_ = −1.02 e Å^−^^3^
Primary atom site location: structure-invariant direct methods	Extinction correction: none

### Special details

**Table:**

Geometry. All e.s.d.'s (except the e.s.d. in the dihedral angle between two l.s. planes) are estimated using the full covariance matrix. The cell e.s.d.'s are taken into account individually in the estimation of e.s.d.'s in distances, angles and torsion angles; correlations between e.s.d.'s in cell parameters are only used when they are defined by crystal symmetry. An approximate (isotropic) treatment of cell e.s.d.'s is used for estimating e.s.d.'s involving l.s. planes.
Refinement. Refinement of *F*^2^ against ALL reflections. The weighted *R*-factor *wR* and goodness of fit *S* are based on *F*^2^, conventional *R*-factors *R* are based on *F*, with *F* set to zero for negative *F*^2^. The threshold expression of *F*^2^ > σ(*F*^2^) is used only for calculating *R*-factors(gt) *etc*. and is not relevant to the choice of reflections for refinement. *R*-factors based on *F*^2^ are statistically about twice as large as those based on *F*, and *R*- factors based on ALL data will be even larger.

### Fractional atomic coordinates and isotropic or equivalent isotropic displacement parameters (Å^2^)

**Table:**

	*x*	*y*	*z*	*U*_iso_*/*U*_eq_	
Pt1	0.659125 (19)	0.633021 (18)	0.106453 (19)	0.02604 (6)	
Cl1	0.77432 (14)	0.58127 (13)	−0.09729 (13)	0.0340 (3)	
S1	0.55837 (15)	0.66420 (15)	0.30825 (14)	0.0370 (3)	
S2	0.48807 (13)	0.75691 (12)	0.03633 (12)	0.0275 (3)	
C1	0.6869 (6)	0.5686 (5)	0.3749 (5)	0.0326 (11)	
C2	0.6782 (7)	0.5500 (6)	0.5051 (6)	0.0412 (14)	
H2	0.5983	0.5897	0.5608	0.049*	
C3	0.7857 (7)	0.4741 (6)	0.5520 (6)	0.0435 (14)	
H3	0.7805	0.4622	0.6401	0.052*	
C4	0.9021 (7)	0.4147 (6)	0.4701 (6)	0.0399 (13)	
H4	0.9763	0.3618	0.5020	0.048*	
C5	0.9085 (6)	0.4330 (5)	0.3449 (6)	0.0343 (12)	
H5	0.9875	0.3931	0.2884	0.041*	
N6	0.8014 (5)	0.5089 (4)	0.2998 (4)	0.0299 (9)	
O7	0.8176 (4)	0.5207 (4)	0.1729 (4)	0.0319 (8)	
O8	0.3857 (4)	0.8369 (4)	0.1274 (4)	0.0389 (10)	
C9	0.5470 (6)	0.8558 (5)	−0.0944 (6)	0.0355 (12)	
H9A	0.4686	0.9050	−0.1282	0.053*	
H9B	0.6103	0.8044	−0.1591	0.053*	
H9C	0.5946	0.9127	−0.0682	0.053*	
C10	0.4004 (6)	0.6700 (6)	−0.0310 (6)	0.0373 (13)	
H10A	0.3568	0.6143	0.0344	0.056*	
H10B	0.4659	0.6197	−0.0955	0.056*	
H10C	0.3300	0.7280	−0.0693	0.056*	
Pt2	0.23052 (2)	−0.015106 (18)	0.666962 (18)	0.02707 (6)	
Cl2	0.36326 (16)	−0.21985 (13)	0.65722 (14)	0.0391 (3)	
S3	0.10091 (15)	0.17934 (13)	0.69804 (14)	0.0340 (3)	
S4	0.23267 (14)	0.00092 (13)	0.46616 (13)	0.0307 (3)	
C11	0.0802 (5)	0.1703 (5)	0.8589 (5)	0.0299 (11)	
C12	−0.0046 (6)	0.2637 (5)	0.9339 (6)	0.0358 (12)	
H12	−0.0528	0.3375	0.8955	0.043*	
C13	−0.0197 (6)	0.2510 (6)	1.0616 (6)	0.0384 (13)	
H13	−0.0784	0.3150	1.1116	0.046*	
C14	0.0521 (6)	0.1429 (6)	1.1175 (6)	0.0376 (13)	
H14	0.0432	0.1331	1.2060	0.045*	
C15	0.1349 (6)	0.0516 (5)	1.0449 (5)	0.0314 (11)	
H15	0.1839	−0.0224	1.0824	0.038*	
N16	0.1469 (4)	0.0670 (4)	0.9177 (4)	0.0272 (9)	
O17	0.2309 (4)	−0.0305 (4)	0.8508 (4)	0.0315 (8)	
O18	0.1467 (5)	0.1149 (4)	0.4162 (4)	0.0430 (10)	
C19	0.4043 (6)	−0.0135 (6)	0.3803 (5)	0.0359 (12)	
H19A	0.4095	−0.0330	0.2943	0.054*	
H19B	0.4645	−0.0812	0.4206	0.054*	
H19C	0.4336	0.0657	0.3784	0.054*	
C20	0.1896 (6)	−0.1328 (6)	0.4240 (6)	0.0377 (13)	
H20A	0.0948	−0.1359	0.4656	0.057*	
H20B	0.2517	−0.2088	0.4502	0.057*	
H20C	0.1985	−0.1279	0.3328	0.057*	

### Atomic displacement parameters (Å^2^)

**Table:**

	*U*^11^	*U*^22^	*U*^33^	*U*^12^	*U*^13^	*U*^23^
Pt1	0.02424 (10)	0.02261 (10)	0.02981 (11)	−0.00326 (7)	−0.00512 (7)	−0.00053 (7)
Cl1	0.0327 (6)	0.0314 (6)	0.0334 (7)	−0.0040 (5)	−0.0012 (5)	−0.0018 (5)
S1	0.0320 (7)	0.0393 (8)	0.0333 (7)	0.0038 (6)	−0.0052 (5)	−0.0030 (6)
S2	0.0264 (6)	0.0248 (6)	0.0306 (6)	−0.0041 (5)	−0.0065 (5)	0.0000 (5)
C1	0.033 (3)	0.028 (3)	0.035 (3)	−0.006 (2)	−0.005 (2)	−0.001 (2)
C2	0.044 (3)	0.042 (3)	0.035 (3)	−0.006 (3)	−0.009 (3)	0.001 (3)
C3	0.051 (4)	0.046 (4)	0.037 (3)	−0.016 (3)	−0.015 (3)	0.007 (3)
C4	0.042 (3)	0.036 (3)	0.046 (3)	−0.012 (3)	−0.020 (3)	0.004 (3)
C5	0.032 (3)	0.027 (3)	0.047 (3)	−0.006 (2)	−0.015 (2)	0.000 (2)
N6	0.031 (2)	0.025 (2)	0.033 (2)	−0.0056 (18)	−0.0087 (18)	0.0007 (18)
O7	0.0277 (18)	0.031 (2)	0.033 (2)	0.0014 (15)	−0.0066 (15)	−0.0017 (16)
O8	0.036 (2)	0.038 (2)	0.037 (2)	0.0083 (17)	−0.0090 (17)	−0.0050 (17)
C9	0.034 (3)	0.025 (3)	0.044 (3)	−0.006 (2)	−0.007 (2)	0.008 (2)
C10	0.035 (3)	0.033 (3)	0.047 (3)	−0.010 (2)	−0.015 (3)	0.003 (2)
Pt2	0.02632 (10)	0.02645 (11)	0.02760 (11)	−0.00455 (8)	−0.00562 (7)	−0.00071 (7)
Cl2	0.0442 (8)	0.0317 (7)	0.0354 (7)	0.0031 (6)	−0.0071 (6)	−0.0041 (5)
S3	0.0385 (7)	0.0286 (7)	0.0342 (7)	−0.0022 (5)	−0.0130 (6)	0.0009 (5)
S4	0.0302 (6)	0.0327 (7)	0.0292 (6)	−0.0079 (5)	−0.0064 (5)	0.0001 (5)
C11	0.027 (2)	0.026 (3)	0.038 (3)	−0.006 (2)	−0.009 (2)	−0.003 (2)
C12	0.032 (3)	0.026 (3)	0.050 (3)	−0.003 (2)	−0.012 (2)	−0.009 (2)
C13	0.034 (3)	0.036 (3)	0.046 (3)	−0.001 (2)	−0.009 (2)	−0.016 (3)
C14	0.034 (3)	0.042 (3)	0.037 (3)	−0.008 (2)	−0.005 (2)	−0.010 (2)
C15	0.029 (3)	0.033 (3)	0.033 (3)	−0.007 (2)	−0.009 (2)	0.000 (2)
N16	0.023 (2)	0.027 (2)	0.031 (2)	−0.0025 (17)	−0.0061 (17)	−0.0047 (17)
O17	0.0299 (19)	0.0267 (19)	0.035 (2)	0.0031 (15)	−0.0077 (15)	−0.0055 (15)
O18	0.043 (2)	0.047 (3)	0.036 (2)	−0.0002 (19)	−0.0122 (18)	0.0021 (19)
C19	0.034 (3)	0.043 (3)	0.032 (3)	−0.015 (2)	−0.003 (2)	−0.003 (2)
C20	0.037 (3)	0.045 (3)	0.038 (3)	−0.018 (3)	−0.009 (2)	−0.007 (3)

### Geometric parameters (Å, °)

**Table:**

Pt1—O7	2.020 (4)	Pt2—O17	2.005 (4)
Pt1—S2	2.1826 (13)	Pt2—S4	2.1850 (14)
Pt1—S1	2.2495 (15)	Pt2—S3	2.2543 (14)
Pt1—Cl1	2.3461 (13)	Pt2—Cl2	2.3402 (14)
S1—C1	1.727 (6)	S3—C11	1.723 (6)
S2—O8	1.475 (4)	S4—O18	1.477 (4)
S2—C9	1.767 (6)	S4—C20	1.767 (6)
S2—C10	1.771 (6)	S4—C19	1.774 (6)
C1—N6	1.346 (7)	C11—N16	1.353 (7)
C1—C2	1.403 (8)	C11—C12	1.394 (8)
C2—C3	1.379 (9)	C12—C13	1.368 (9)
C2—H2	0.95	C12—H12	0.95
C3—C4	1.394 (10)	C13—C14	1.396 (9)
C3—H3	0.95	C13—H13	0.95
C4—C5	1.353 (9)	C14—C15	1.359 (8)
C4—H4	0.95	C14—H14	0.95
C5—N6	1.366 (7)	C15—N16	1.366 (7)
C5—H5	0.95	C15—H15	0.95
N6—O7	1.358 (6)	N16—O17	1.378 (5)
C9—H9A	0.98	C19—H19A	0.98
C9—H9B	0.98	C19—H19B	0.98
C9—H9C	0.98	C19—H19C	0.98
C10—H10A	0.98	C20—H20A	0.98
C10—H10B	0.98	C20—H20B	0.98
C10—H10C	0.98	C20—H20C	0.98
			
O7—Pt1—S2	179.05 (12)	O17—Pt2—S4	179.35 (11)
O7—Pt1—S1	86.29 (11)	O17—Pt2—S3	86.41 (11)
S2—Pt1—S1	93.15 (5)	S4—Pt2—S3	94.02 (5)
O7—Pt1—Cl1	89.02 (11)	O17—Pt2—Cl2	87.69 (11)
S2—Pt1—Cl1	91.57 (5)	S4—Pt2—Cl2	91.89 (5)
S1—Pt1—Cl1	174.53 (5)	S3—Pt2—Cl2	174.07 (5)
C1—S1—Pt1	97.4 (2)	C11—S3—Pt2	97.42 (19)
O8—S2—C9	107.7 (3)	O18—S4—C20	108.9 (3)
O8—S2—C10	108.1 (3)	O18—S4—C19	108.9 (3)
C9—S2—C10	101.3 (3)	C20—S4—C19	101.1 (3)
O8—S2—Pt1	116.54 (17)	O18—S4—Pt2	117.14 (19)
C9—S2—Pt1	111.3 (2)	C20—S4—Pt2	110.2 (2)
C10—S2—Pt1	110.8 (2)	C19—S4—Pt2	109.4 (2)
N6—C1—C2	117.9 (5)	N16—C11—C12	117.2 (5)
N6—C1—S1	119.2 (4)	N16—C11—S3	119.3 (4)
C2—C1—S1	122.9 (5)	C12—C11—S3	123.5 (4)
C3—C2—C1	119.9 (6)	C13—C12—C11	121.3 (6)
C3—C2—H2	120.1	C13—C12—H12	119.4
C1—C2—H2	120.1	C11—C12—H12	119.4
C2—C3—C4	119.9 (6)	C12—C13—C14	119.2 (5)
C2—C3—H3	120.0	C12—C13—H13	120.4
C4—C3—H3	120.0	C14—C13—H13	120.4
C5—C4—C3	119.4 (6)	C15—C14—C13	119.8 (6)
C5—C4—H4	120.3	C15—C14—H14	120.1
C3—C4—H4	120.3	C13—C14—H14	120.1
C4—C5—N6	120.0 (6)	C14—C15—N16	119.4 (5)
C4—C5—H5	120.0	C14—C15—H15	120.3
N6—C5—H5	120.0	N16—C15—H15	120.3
C1—N6—O7	121.7 (4)	C11—N16—C15	123.0 (5)
C1—N6—C5	122.9 (5)	C11—N16—O17	120.9 (4)
O7—N6—C5	115.4 (5)	C15—N16—O17	116.0 (4)
N6—O7—Pt1	115.3 (3)	N16—O17—Pt2	115.4 (3)
S2—C9—H9A	109.5	S4—C19—H19A	109.5
S2—C9—H9B	109.5	S4—C19—H19B	109.5
H9A—C9—H9B	109.5	H19A—C19—H19B	109.5
S2—C9—H9C	109.5	S4—C19—H19C	109.5
H9A—C9—H9C	109.5	H19A—C19—H19C	109.5
H9B—C9—H9C	109.5	H19B—C19—H19C	109.5
S2—C10—H10A	109.5	S4—C20—H20A	109.5
S2—C10—H10B	109.5	S4—C20—H20B	109.5
H10A—C10—H10B	109.5	H20A—C20—H20B	109.5
S2—C10—H10C	109.5	S4—C20—H20C	109.5
H10A—C10—H10C	109.5	H20A—C20—H20C	109.5
H10B—C10—H10C	109.5	H20B—C20—H20C	109.5
			
O7—Pt1—S1—C1	−0.8 (2)	O17—Pt2—S3—C11	−5.7 (2)
S2—Pt1—S1—C1	180.0 (2)	S4—Pt2—S3—C11	174.82 (19)
S1—Pt1—S2—O8	12.4 (2)	S3—Pt2—S4—O18	−5.8 (2)
Cl1—Pt1—S2—O8	−170.4 (2)	Cl2—Pt2—S4—O18	173.6 (2)
S1—Pt1—S2—C9	136.5 (2)	S3—Pt2—S4—C20	−131.0 (2)
Cl1—Pt1—S2—C9	−46.3 (2)	Cl2—Pt2—S4—C20	48.5 (2)
S1—Pt1—S2—C10	−111.7 (2)	S3—Pt2—S4—C19	118.7 (2)
Cl1—Pt1—S2—C10	65.5 (2)	Cl2—Pt2—S4—C19	−61.8 (2)
Pt1—S1—C1—N6	1.1 (5)	Pt2—S3—C11—N16	4.8 (4)
Pt1—S1—C1—C2	−179.1 (5)	Pt2—S3—C11—C12	−174.0 (5)
N6—C1—C2—C3	0.9 (9)	N16—C11—C12—C13	−0.1 (8)
S1—C1—C2—C3	−178.9 (5)	S3—C11—C12—C13	178.7 (5)
C1—C2—C3—C4	−0.8 (10)	C11—C12—C13—C14	0.5 (9)
C2—C3—C4—C5	0.4 (10)	C12—C13—C14—C15	−0.5 (9)
C3—C4—C5—N6	−0.1 (9)	C13—C14—C15—N16	0.2 (9)
C2—C1—N6—O7	179.4 (5)	C12—C11—N16—C15	−0.3 (8)
S1—C1—N6—O7	−0.9 (7)	S3—C11—N16—C15	−179.1 (4)
C2—C1—N6—C5	−0.7 (8)	C12—C11—N16—O17	178.9 (5)
S1—C1—N6—C5	179.1 (4)	S3—C11—N16—O17	0.0 (7)
C4—C5—N6—C1	0.3 (9)	C14—C15—N16—C11	0.2 (8)
C4—C5—N6—O7	−179.7 (5)	C14—C15—N16—O17	−178.9 (5)
C1—N6—O7—Pt1	0.0 (6)	C11—N16—O17—Pt2	−5.9 (6)
C5—N6—O7—Pt1	−180.0 (4)	C15—N16—O17—Pt2	173.3 (4)
S1—Pt1—O7—N6	0.6 (3)	S3—Pt2—O17—N16	7.0 (3)
Cl1—Pt1—O7—N6	−176.6 (3)	Cl2—Pt2—O17—N16	−172.5 (3)

### Hydrogen-bond geometry (Å, °)

**Table:**

*D*—H···*A*	*D*—H	H···*A*	*D*···*A*	*D*—H···*A*
C9—H9A···O17^i^	0.98	2.43	3.351 (8)	157
C9—H9B···Cl1	0.98	2.73	3.350 (6)	121
C10—H10A···Cl1^ii^	0.98	2.73	3.601 (7)	149
C13—H13···O7^iii^	0.95	2.37	3.268 (8)	158
C15—H15···O8^iv^	0.95	2.39	3.271 (7)	155
C19—H19A···O8^v^	0.98	2.54	3.456 (7)	155
C20—H20A···O18^vi^	0.98	2.49	3.460 (8)	172
C20—H20B···Cl2	0.98	2.75	3.366 (7)	122

Symmetry codes: (i) *x*, *y*+1, *z*−1; (ii) −*x*+1, −*y*+1, −*z*; (iii) *x*−1, *y*, *z*+1; (iv) *x*, *y*−1, *z*+1; (v) *x*, *y*−1, *z*; (vi) −*x*, −*y*, −*z*+1.

## References

[bb1] Allen, F. H., Kennard, O., Watson, D. G., Brammer, L., Orpen, A. G. & Taylor, R. (1987). *J. Chem. Soc. Perkin Trans. 2*, pp. S1–S19.

[bb2] Bovin, D. H. R., Crepon, E. & Zard, S. Z. (1992). *Bull. Soc. Chem. Fr.* **129**, 145–150.

[bb3] Bruker (2006). *APEX2* and *SAINT* Bruker AXS Inc., Madison, Wisconsin, USA.

[bb16] Coppens, P. (1970). *Crystallographic Computing*, edited by F. R. Ahmed, S. R. Hall & C. P. Huber, pp. 255–270. Copenhagen: Munksgaard.

[bb4] Dyksterhouse, R. M., Howell, B. A. & Squattrito, P. J. (2000). *Acta Cryst.* C**56**, 64–66. 10.1107/s0108270199013803 10710671

[bb5] Jebas, S. R., Balasubramanian, T., Ravidurai, B. & Kumaresan, S. (2005). *Acta Cryst.* E**61**, o2677–o2678.

[bb6] Katsuyuki, N., Carter, B. J., Xu, J. & Hetch, S. M. (1991). *J. Am. Chem. Soc.* **113**, 5099–5100.

[bb7] Leonard, F., Barklay, F. A., Brown, E. V., Anderson, F. E. & Green, D. M. (1955). *Antibiot. Chemother.* pp. 261–264. 24543958

[bb8] Lobana, T. S. & Bhatia, P. K. (1989). *J. Sci. Ind. Res.* **48**, 394–401.

[bb9] Loehrer, P. J., William, S. D. & Einhorn, L. H. (1988). *J. Natl Cancer Inst.* **80**, 1373–1376. 10.1093/jnci/80.17.1373 3050140

[bb10] Ohms, U., Guth, H., Kutoglu, A. & Scheringer, C. (1982). *Acta Cryst.* B**38**, 831–834.

[bb11] Ravindran Durai Nayagam, B., Jebas, S. R., Grace, S. & Schollmeyer, D. (2008). *Acta Cryst.* E**64**, o409. 10.1107/S1600536807068766 PMC2960153 21201437

[bb12] Sheldrick, G. M. (2008). *Acta Cryst.* A**64**, 112–122. 10.1107/S0108767307043930 18156677

[bb13] Spek, A. L. (2003). *J. Appl. Cryst.* **36**, 7–13.

[bb14] Symons, M. C. R. & West, D.-X. (1985). *J. Chem. Soc. Dalton Trans.* pp. 379–381.

[bb15] Weiss, R. B. & Christian, M. C. (1993). *Drugs*, **46**, 360–377. 10.2165/00003495-199346030-00003 7693428

